# Mating system variation in hybrid zones: facilitation, barriers and asymmetries to gene flow

**DOI:** 10.1111/nph.16180

**Published:** 2019-10-09

**Authors:** Melinda Pickup, Yaniv Brandvain, Christelle Fraïsse, Sarah Yakimowski, Nicholas H. Barton, Tanmay Dixit, Christian Lexer, Eva Cereghetti, David L. Field

**Affiliations:** ^1^ Institute of Science and Technology Austria Am Campus 1 Klosterneuburg 3400 Austria; ^2^ Department of Plant and Microbial Biology University of Minnesota 1500 Gortner Ave St Paul, Minneapolis MN 55108 USA; ^3^ Department of Biology Queen's University 116 Barrie St Kingston ON K7L 3N6 Canada; ^4^ Department of Zoology University of Cambridge Downing Street Cambridge CB2 3EJ UK; ^5^ Department of Botany and Biodiversity Research Faculty of Life Sciences University of Vienna A‐1030 Vienna Austria; ^6^ School of Science Edith Cowan University 270 Joondalup Drive Joondalup Western Australia 6027 Australia

**Keywords:** gene flow, hybridization, introgression, mating system, reproductive isolation

## Abstract

Plant mating systems play a key role in structuring genetic variation both within and between species. In hybrid zones, the outcomes and dynamics of hybridization are usually interpreted as the balance between gene flow and selection against hybrids. Yet, mating systems can introduce selective forces that alter these expectations; with diverse outcomes for the level and direction of gene flow depending on variation in outcrossing and whether the mating systems of the species pair are the same or divergent. We present a survey of hybridization in 133 species pairs from 41 plant families and examine how patterns of hybridization vary with mating system. We examine if hybrid zone mode, level of gene flow, asymmetries in gene flow and the frequency of reproductive isolating barriers vary in relation to mating system/s of the species pair. We combine these results with a simulation model and examples from the literature to address two general themes: (1) the two‐way interaction between introgression and the evolution of reproductive systems, and (2) how mating system can facilitate or restrict interspecific gene flow. We conclude that examining mating system with hybridization provides unique opportunities to understand divergence and the processes underlying reproductive isolation.

## Introduction

For plant mating, the relationship between parents varies tremendously: ranging from within the same individual (selfing) at one extreme, to interspecific hybridization on the other. Studies of hybrid zones, where reproductive isolation is incomplete, examine one end of this continuum, offering windows into the speciation process and the maintenance of divergence in sympatry (Barton & Hewitt, 1985; Abbott *et al*., 2013; Gompert *et al*., 2017). At the other extreme, studies of mating systems examine the frequency and consequences of mating among relatives. This offers insight into the evolution of genetic load, the distribution of genetic variation within and among populations, and the extent of intragenomic conflicts (Brandvain & Haig, 2005; Glémin *et al*., 2006; Hough *et al*., 2013). The research fields of hybridization and mating systems have developed largely independently, even though they address complementary questions regarding the costs and benefits of inbreeding and outcrossing. While some studies have considered this interaction, there is still no overall synthesis of how different mating/sexual system combinations may influence the occurrence and outcomes of hybridization. Moreover, studying hybrid zones in the context of different mating systems opens new avenues for understanding both the speciation process and the evolution of mating systems and their consequences.

In flowering plants, hybrid zones – regions of contact between divergent populations with individuals of mixed ancestry (Barton & Hewitt, 1985) – have been reported across a broad range of taxa, life‐histories and biogeographic regions (Abbott, 2017). Hybrid zones can illuminate the pre‐ and post‐zygotic factors that limit interspecific gene flow (Rieseberg & Carney, 1998; Abbott *et al*., 2013). In plants, pre‐mating pre‐zygotic barriers act before pollen deposition and can include differences in geographic distributions, habitat, phenology and pollinators. Once pollen is transferred between species, pollen–pistil interactions may limit pollen tube growth in one or both directions (post‐mating pre‐zygotic barriers). Post‐zygotic barriers may include hybrid inviability or sterility due to intrinsic incompatibilities, and reduced hybrid fitness relative to parental genotypes due to maladaptation to the extrinsic environment. When hybrids are formed, the rate of gene flow (dispersal) relative to the strength of selection against hybrids shapes hybrid zones (Barton & Gale, 1993). Thus, identifying the forces that mediate the rate and direction of gene flow is central to understanding reproductive barriers and the evolutionary fate of diverging lineages. Here we argue that examining variation in plant mating systems provides a valuable opportunity to understand the forces underlying gene flow in hybridizing species‐pairs.

**Figure 1:**
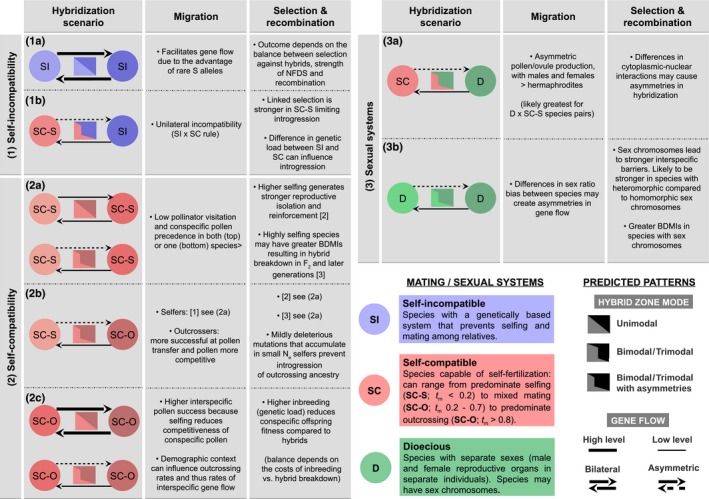
The potential influence of different mating/sexual systems on hybridization patterns. The different sections follow the main text: (1) self‐incompatible (SI) systems and the SI × SC rule, (2) self‐compatible systems (selfers, outcrossers and mixed‐maters, SC‐O in the figure is equivalent to SC‐OC in the main text) and (3) sexual systems (dioecy with or without sex chromosomes). On the left‐hand side of each panel, hybridization scenarios are depicted. For each scenario, the two coloured circles represent the mating/sexual system of each taxon, which can affect hybrid zone mode (rectangle) and the level and direction of gene flow (arrows). Possible underlying processes are shown on the middle panel (migration) and on the right‐hand side (selection and recombination). BDMIs, Bateson–Dobzhansky–Muller incompatibilities; S alleles, self‐incompatibility alleles; NFDS, negative frequency dependent selection; *t*_m_, outcrossing rate.

The rich diversity of mating and sexual systems in plants is an important source of variation influencing patterns of dispersal and selection across genomes. The proportion of cross‐fertilization may vary considerably among individuals and populations (mixed‐maters) whereas others maintain high levels of outcrossing due to genetic self‐incompatibility (Goodwillie *et al*., 2005; Charlesworth, 2006). In other groups, selfing avoidance has likely driven the evolution of separate sexes (dioecy) and related gender strategies (gynodioecy, androdioecy) or stylar polymorphisms (e.g. heterostyly) (Charlesworth, 2006; Barrett, 2010). Given their diversity, and that mating systems differ in how they influence gene transfer, each mating system type may interact uniquely with hybridization (Fig. 1). Bringing together the literature on hybridization and mating systems can offer important insights into their interaction and how this may impact the evolution of both mating systems and reproductive isolation. More generally, the differences associated with alternative plant mating systems provide an opportunity to test how features including genetic conflicts (Brandvain & Haig, 2005; Sweigart *et al*., 2019), rare‐advantage (Bierne *et al*., 2002), and genetic load (Kim *et al*., 2018) promote or inhibit gene flow. Studying the interaction of mating systems and speciation can illuminate biological questions that are actively debated in taxa whose limited variation in mating system precludes such investigations.

Here, we examine the evolutionary consequences of hybridization between plant species pairs with similar and contrasting mating systems. We first consider each of the major mating system types and combinations observed in plant hybrid zones and highlight the potential influence of these on gene flow, selection and recombination. We also present predictions of how different mating systems may affect hybrid zone mode and level of gene flow (Fig. 1; Supporting Information Table S1). Next we present a literature survey of 133 plant hybrid zone studies to examine how patterns of hybridization vary with mating system, and to test predictions for specific mating system combinations. We then use a simulation model and individual case studies to highlight how different mating system combinations influence rates and asymmetries in gene flow in hybrid zones. Finally, we conclude by discussing open questions and future directions (Box 1) and how – by combining mating system and hybridization – we can better understand divergence and the processes underlying reproductive isolation between divergent mating strategies.

Box 1 **How does mating system interact with hybridization: open questions and future directions**

A unifying feature of all mating system combinations in hybridizing species pairs is the variety of mechanisms that can influence the key drivers of hybrid zone dynamics: gene flow, selection and recombination (see Fig. 1). Yet it is this complexity that both generates novel questions and highlights the current lack of data to test specific hypotheses. We outline a number of open questions and the data required to test these in hybridizing taxa. Overall, we emphasize the need for data that enables the effects of mating system to be considered while controlling for divergence time between species.

**Molecular genetics, phylogenetics and ecology**

**Are mating system combinations represented across all hybridizing species groups?**

Our comparative data revealed that most plant hybrid zones involve the interaction between the same mating system type (SC × SC or SI × SI; self‐compatible (SC), self‐incompatible (SI)). This is unlikely a random sample of hybridizing taxa because studies of hybrid zones and mating systems are often motivated by different questions. More information on the distributional range of each taxa will help to distinguish whether lack of hybrid zones between some divergent mating system classes is due to very strong barriers (i.e. no hybrids form), a lack of geographic overlap or limited variation in mating systems within some genera.

**How do outcrossing rates and mating patterns vary among different mating system combinations? How does this interact with ecology and demography?**

Given the potential for selfing rates to mediate opportunities for hybridization, data on outcrossing rates and mating patterns across replicate hybrid zones would allow connection of local ecology/demography, phenology and morphological traits (e.g. degree of herkogamy, cleistogamy/chasmogamy). This would allow for a greater understanding of the role of the environment vs genetic variation in mediating mating patterns and hybridization. Likewise, in strictly dioecious (D) species, sex ratio bias can vary dramatically (Field *et al*., 2013), and stronger sex ratio bias in one species may generate asymmetrical gene flow across nuclear and cytoplasmic genomes (Currat *et al*., 2008). To address this gap, sex ratio data is required for D hybrid zones to assess its potential influence on gene flow.

**Do reproductive isolating barriers vary among taxa with different mating system combinations?**

Given the cumulative nature of reproductive isolating barriers (Lowry *et al*., 2008), the importance of mating system for hybridization will depend on when the barrier to gene exchange acts. The effect of mating system on pre‐zygotic barriers is likely to be more important for reproductive isolation (and overall barrier strength) than post‐zygotic ones (hybrid viability and sterility). To address these questions, studies that quantify components of reproductive isolation for different mating system combinations are required.

**Genomic data**

**How does mating system influence the effect of linked selection on the genomic landscape of introgression?**

By driving the probability that neutral and incompatible alleles are uncoupled, recombination rate can influence the degree of introgression over long timescales. This leads to the expectation of a positive correlation between introgression and recombination rate (e.g. Schumer *et al*., 2018; Martin *et al*., 2019) which is a relation that may be mediated by mating system. Methods from molecular evolution can provide further insight into the evolutionary forces that shape species barriers. The McDonald–Kreitman framework can be used to estimate what fraction of mutations were fixed between species due to adaptive divergent selection (e.g. Christe *et al*., 2017; Rifkin *et al*., 2019), and test whether this varies across mating systems.

**Theory and simulations**

**Methodological challenges of nonrandom mating**

Identifying interspecific barriers to hybridization is a challenging task, as factors such as complex demography and selection at linked sites can mimic signatures of divergent selection. Methods must therefore account for these confounding factors in null models to accurately uncover signatures of divergent selection. Mating systems – such as selfing – add a layer of complexity as most tools are not designed to model nonrandom mating. Consequently, new methods are required that estimate the relevant genetic parameters applicable to different mating systems. For example, future research could follow Rifkin *et al*. (2019), who developed a method to infer admixture contributions (and asymmetries) in the presence of selfing.

**What is the effect of mating system on hybridization rates and outcomes for different mating system combinations?**

Our comparative analysis generates many predictions on how mating system may affect gene flow patterns in hybrid zones. Yet few of these verbal models have been formally evaluated by theory (but see Epinat & Lenormand, 2009; Hu, 2015; Rausher, 2016). Consequently, the biological conditions and timescales under which they apply remain largely unclear. To begin filling this gap, our simulation study uncovered the interplay between SI and selection against hybrids, and demonstrated that SI can facilitate introgression under strong negative frequency‐dependent selection. Further work could address both the short‐ and long‐term dynamics of hybridization between different mating system combinations.

## Predictions on the influence of mating systems on hybridization

The evolutionary consequences of hybridization between taxa will vary depending on whether the taxa have the same or divergent mating systems. First, we consider self‐incompatibility, where strong balancing selection is expected to facilitate gene flow leading to a tension between hybrid inviability and rare allele advantage (Fig. 1, section 1). Second, we consider hybridization between self‐compatible (SC) taxa, which may vary in the degree of selfing vs outcrossing (Fig. 1, section 2). For highly selfing taxa, low hybridization rates are expected due to reduced pollen transfer and stronger reproductive isolation. By contrast, strong asymmetries in gene flow and barrier strength are expected for hybridization between selfing and outcrossing systems. Differences in the strength of linkage disequilibrium across stretches of the genome in selfers and outcrossers may also influence how advantageous and disadvantageous blocks of genome disperse through hybrid zones. For mixed maters (variable outcrossing rates), rates of hybridization and gene flow may change depending on ecological and/or demographic context (Fig. 1, section 2). Third, we consider hybridization between different sexual systems such as between two D species (with separate sexes) or between D and SC (hermaphrodite) taxa (Fig. 1, section 3). For these species pairs, differences in reproductive strategies can influence barrier strength and result in asymmetrical gene flow. Moreover, sex chromosomes and cyto‐nuclear interactions may generate stronger barriers, resulting in restricted hybridization and reduced gene flow.

## Comparative analysis: mating systems can affect gene flow and asymmetries in hybridization

**Figure 2:**
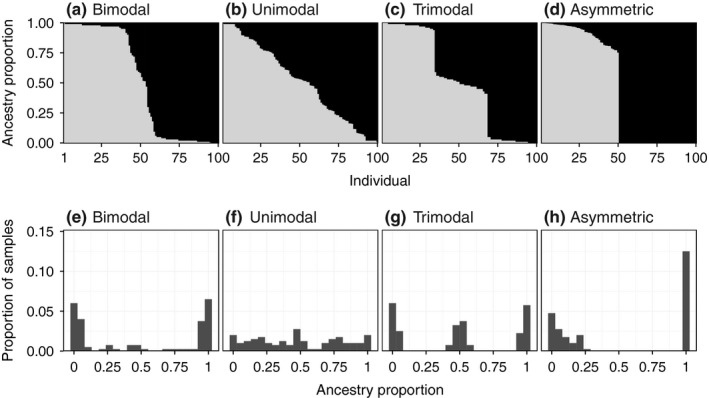
Hybrid zones are defined as geographic regions of genetic mixing between divergent populations. Hybrid indices and admixture coefficients tell us about the proportion of an individual's genome inherited from one or the other parental species. In this example, the same individual ancestry proportions are represented in (a–d) ordered by the proportion of ancestry belonging to parental species A and in (e–h) as a frequency distribution. This distribution in a hybrid zone may reflect the strength and direction of reproductive barriers. In strongly bimodal hybrid zones (a, e) few hybrids are present and pure parental genotypes dominate, suggesting a strong barrier with very low levels of gene exchange. By contrast, for unimodal hybrid zones (b, f) the distribution spans a range of admixture and backcrosses toward both parents, which suggests weaker barriers and high levels of gene exchange. In trimodal hybrid zones (c, g) high frequencies of F_1_s and limited backcrosses may suggest F_1_ sterility and/or heterosis. Highly skewed distributions with backcrossing predominantly to one parental type (d, h) may reflect the direction of gene flow and imply asymmetrical strength in reproductive barriers.

To investigate the interaction between hybridization and mating system we collated data on hybridization (see Fig. 2) in 133 species pairs, representing 72 genera and 41 plant families (for methods see Methods S1). Mating/sexual system classifications included: self‐incompatible (SI), self‐compatible (SC), dioecious (D), gynodioecious (G), androdioecious (A) and trioecious (T) (for a description of each system type see Table S1). We further classified species capable of self‐fertilization (self‐compatible, SC) into predominantly selfing (SC‐S) and predominantly outcrossing (including mixed maters, SC‐OC) based on outcrossing rates (*t*_m_) (where available) and descriptions from the literature (SC‐S: *t*_m_ ≤ 0.2, SC‐OC: *t*_m_ > 0.2).

**Figure 3:**
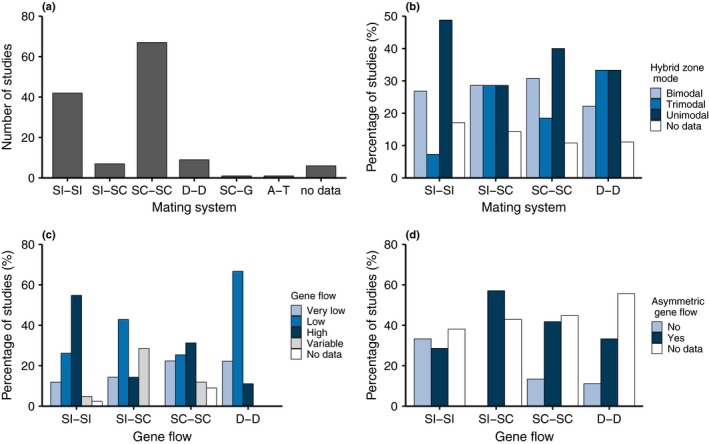
Comparative analysis for 133 angiosperm species pairs by mating system combination (see Supporting Information Table S1 for descriptions of each mating system type). (a) The number of species pairs in each mating system combination: both self‐incompatible (SI × SI,* n* = 42), self‐incompatible × self‐compatible (SI × SC,* n* = 7), both self‐compatible (SC × SC,* n* = 67), both dioecious (D × D, *n* = 9), self‐compatible × gynodioecious (SC × G, *n* = 1), androdioecious × trioecious (A × T, *n* = 1) and unknown (mating system of one or both taxa unknown, *n* = 6). (b) Proportion of species pairs classified as each hybrid zone mode (bimodal, trimodal and unimodal). (c) Proportion of species pairs categorized by level of gene flow (very low, low, high and variable). (d) Proportion of species pairs that recorded asymmetries in gene flow.

Both hybridizing taxa were SC in half (*n* = 67, 52.8%) of the 127 species pairs with data for both parental taxa, followed by pairs where both taxa were SI (*n* = 42, 33.1%) and both D (*n* = 9, 7.1%) (Fig. 3a). Fewer species pairs included taxa with different mating/sexual systems, seven (5.6%) between self‐incompatible and self‐compatible taxa (SI × SC), one between a self‐compatible and gynodioecious species, and another between an androdioecious and trioecious species (Fig. 3a). For hybridization between two SC species (*n* = 67), the majority (*n* = 56, 83.5%) were between outcrossing taxa (SC‐OC × SC‐OC), with four cases of hybridization between highly selfing taxa (SC‐S × SC‐S) and seven between a predominant outcrosser and selfer (SC‐S × SC‐OC). For six species pairs, one or both mating systems were unknown (Fig. 3a). There was a significant excess of hybrid zones between species pairs with like mating systems (*χ* ^2^ = 15.09, simulation‐based *P *=* *0.0027). However, with this data it was not possible to resolve whether this difference reflects a biological propensity for such zones to be formed, or phylogenetic nonindependence. Our survey is based on the best available data from published studies. Yet it is well known that selection of plant species pairs for study is not random, which may result in a bias for global estimates of mating system frequency and/or hybridization rate. Nonetheless, we found no systematic bias with mating system. For example, although the extent of gene flow is likely overestimated (because hybrid zones in species with little to no hybridization are rarely studied), this bias did not differ by mating systems of the parental species.

Genetic characterization of hybrid zones using a wide range of genetic markers (allozymes, RFLPs, AFLPs, SSRs, SNPs) and numbers of loci (*n* = 4–1000s) precluded quantitative comparisons on the same scale. Therefore, we used hybrid zone mode (unimodal, bimodal and trimodal), as a qualitative measure that reflects the genotypic composition of hybrid zones and provides information on the strength of reproductive barriers (see Fig. 2). Unimodal hybrid zones indicate ongoing hybridization and admixture, with the presence of a range of hybrid admixture types; whereas bimodal (predominantly parental genotypes and low‐frequency hybrids) and trimodal (predominantly parents and F_1_ hybrids) are indicative of stronger reproductive barriers.

The frequency of hybrid zone mode did not vary significantly among the four main mating system types (*χ* ^2^ = 9.5, permutation based *P *=* *0.7950, Fig. 3b). Restricting to SC × SC and SI × SI species pairs with unimodal, bimodal or trimodal hybrid zones (92 of our 111 data points), we still found no significant association between hybrid zone type and mating system combination (*χ* ^2^ = 2.7, df = 2, *P *=* *0.2567). Unimodal hybrid zones were the most common in this data set, making up half of the 92 observations. Bimodal hybrid zones were also common, making up *c*. 1/3 of the hybrid zones (31 of 92). Trimodal hybrid zones where least common, representing *c*. 1/6 of the hybrid zones (15 of 92).

Using a qualitative classification for the level of gene flow (‘high’ vs ‘low variable, low, or very low’; see Methods S1) we found that the distribution of gene flow categories varied with mating system type (*χ* ^2^ = 10.316, df = 3, *P *=* *0.0161, with mating system combinations D–D, SC–SC, SI–SC, SI–SI, Fig. 3c). This difference is primarily attributable to the excess of SI × SI hybrid zones with high gene flow (23 of 41). High gene flow was much less common in hybrid zones between SC species, making up 21 of the 61 characterized cases. High gene flow was even rarer in SI × SC (one of seven), and D × D (one of nine) hybrid zones. The single A × T species pair with known levels of gene flow was characterized as low‐variable, while the lone characterized SC–G hybrid zone had a low level of gene flow.

Asymmetries in gene flow were present for all mating system types (Fig. 3d), and quite common – comprising 49 of the 73 cases for which asymmetry was evaluated. The frequency of asymmetry varied by mating system (*χ* ^2^ = 8.12, df = 2, *P *=* *0.0172) among the 67 SC–SC, SI–SC, SI–SI hybrid zones for which asymmetry was characterized. Asymmetric gene flow was predominant in SC × SC pairs (28 of the 37), and all four SI × SC hybrid zones for which such data was available. In comparison, SI × SI hybrid zones had comparable numbers of symmetric (*n* = 14) and asymmetric (*n* = 12) pairs.

Given the categorical nature of hybrid zone mode and gene flow level, we use the numbers in each category to describe general trends and make predictions for each mating system type (see Fig. 1). We now discuss these results in the context of the broader literature on mating systems and hybridization for three main types: (1) self‐incompatibility (SI × SI, SI × SC), (2) self‐compatibility (SC × SC, including hybridization between predominant selfers (SC‐S × SC‐S), mixed maters (SC‐OC × SC‐OC) and between selfers and outcrossers (SC‐S × SC‐OC) and (3) sexual system variation (including hybridization between dioecious species (D × D) and between dioecious and self‐compatible species (D × SC)).

## Self‐incompatibility

For genetically based self‐incompatibility systems (both self‐ and nonself‐recognition; for review see Fujii *et al*., 2016), mating can only occur among plants with different alleles at the self‐incompatibility locus. The high allelic diversity observed in these systems (see Lawrence, 2000) is maintained by negative frequency‐dependent selection (a form of balancing selection) where rare alleles have a high selective advantage (Wright, 1939). For species with weak reproductive barriers, outcrossing mechanisms such as SI can influence the rate of hybridization (Ellstrand *et al*., 1996) and its evolutionary outcomes. Self‐incompatibility can facilitate gene flow at the S locus (Castric *et al*., 2008), impeding speciation and increasing rates of introgression, while selection against hybrids may restrict introgression. Consequently, the balance between gene flow and selection determines the role of mating system in promoting hybridization. High and symmetric levels of gene flow were frequently observed in hybrid zones of two SI species (Fig. 3c,d). Yet, the cases of lower gene flow may reflect greater hybrid breakdown, which may counteract the increase in migration rate associated with S allele exchange (see simulation model below), leading to an overall reduction in gene flow.

### Self‐incompatibility alleles can facilitate gene flow: a simulation model

Understanding the potential influence of self‐incompatibility on rates of introgression requires examination of the influence of both the S locus (locus determining self‐incompatibility) and selection against hybrids (barrier locus) on the outcomes of hybridization. For the S locus, negative frequency‐dependent selection results in a selective advantage for migrants with rare alleles, which may increase the effective migration rate. Conversely, the presence of a genetic barrier due to reduced hybrid fitness may reduce effective migration rate via selection against foreign alleles (Barton & Bengtsson, 1986). These two forces should act in opposing directions, with the S locus facilitating, while hybrid breakdown preventing, introgression. Yet, the relative strength of these two forces will depend on parameters such as S allele diversity, degree of population differentiation, the strength of selection against hybrids and genetic architecture. To investigate if self‐incompatibility can counteract selection against hybrids, resulting in greater introgression, we simulated populations of two demes with both self‐incompatibility and a barrier locus (for model description see Methods S2). Our model varied the number of S alleles, sharing of S alleles between demes, strength of selection against hybrids, and recombination rates between the barrier locus and the neutral marker to ask: How does population structure (differentiation) in S alleles influence introgression? How does introgression vary with S allele diversity and the strength of selection against hybrids? How does linkage affect the balance between the benefit of novel S alleles and costs of hybrid breakdown?

**Figure 4:**
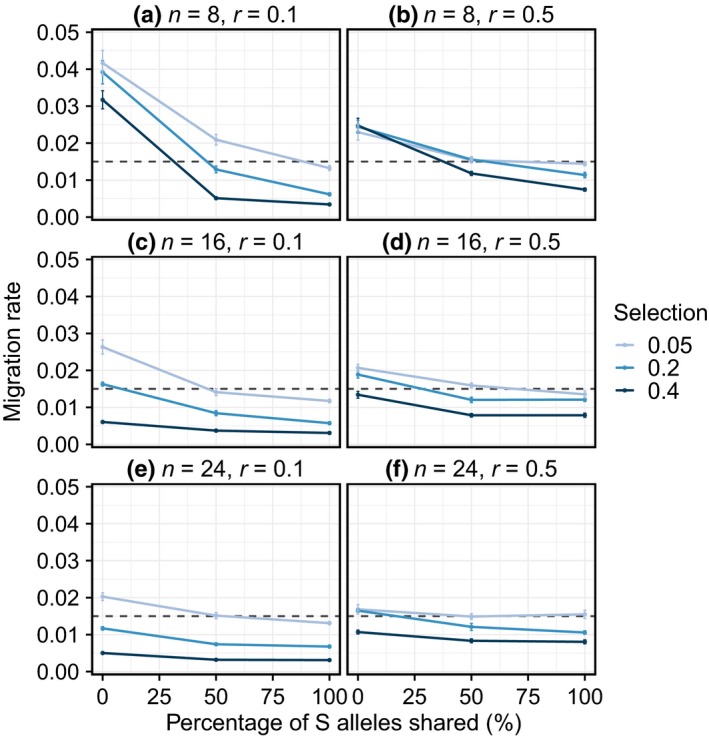
The effect of varying the strength of selection (*s* = 0.05, 0.2, 0.4) and the number (*n* = 8, 16, 24) and percentage sharing (none, 50%, 100%) of S alleles on effective migration rate. Panels (a, c, e) are for a recombination rate (*r*) of 0.1 between the barrier locus (hybrid unfitness) and the neutral locus used to estimate effective migration rate, while (b, d, f) are when *r* = 0.5, representing no physical linkage between the neutral and barrier locus. Figures show means ± SE for 20 replicates of each combination of parameters. Effective migration rate was measured at a neutral locus over the first 25 generations (see Supporting Information Methods S2). The dashed line shows the actual migration rate (*m* = 0.015).

Under certain conditions, self‐incompatibility can overcome even strong selection against hybrids, resulting in a higher effective migration rate (Fig. 4). The effective migration rate was highest when demes had unique sets of S alleles (none shared), there was weak selection against hybrids (*s* = 0.05) and few S alleles (*n* = 8) (Fig. 4a). With eight S alleles, under maximum S allele differentiation (none shared), the effective migration rate among demes was high even with strong selection against hybrids (*s* = 0.4, Fig. 4a). Yet when demes contained 16 and 24 S alleles, the elevated effective migration rate was only observed with weak selection against hybrids (Fig. 4c,e). With higher numbers of S alleles (*n* = 16 and 24) and strong selection at the barrier locus, effective migration rate was reduced below the actual migration rate when demes shared half or all S alleles (Fig. 4c,e). We found that linkage between the locus that reduced hybrid fitness (barrier locus) and the neutral locus strengthened the effects of both the S locus and the barrier locus on effective migration rate. For example, under strong negative frequency‐dependent selection (*n* = 8, no shared S alleles), effective migration rate was higher with linkage (Fig. 4a) compared to unlinked loci (*r* = 0.5, Fig. 4b). Similarly, with stronger selection against hybrids (*s* = 0.2, 0.4), linkage (*r* = 0.1) reduced the effective migration rate further with both 16 (Fig. 4c,d) and 24 (Fig. 4e,f) S alleles.

Our model illustrates that under conditions associated with strong negative frequency‐dependent selection (low diversity and high differentiation in S alleles), self‐incompatibility can facilitate gene flow and overcome the effects of strong selection against hybrids. The higher effective migration rate under these conditions could facilitate introgression, even with barriers to gene flow. This is a transient phenomenon, as populations reach equilibrium and share S alleles, which will reduce the strength of negative frequency‐dependent selection. However, the stochastic loss of S alleles could re‐establish the conditions that facilitate introgression.

Tension between genomic regions underlying self and interspecific incompatibility depends on the underlying genetic architecture of the selected loci (Barton & Hewitt, 1985). The current model focuses on a simple two‐locus model with one locus controlling hybrid fitness (barrier locus), which would reflect the situation where loci of major effect determine hybrid fitness, such as flower colour (e.g. Tavares *et al*., 2018). Extending this model to include more selected loci would more likely reflect the genetic architecture of hybrid fitness for divergent species. Linkage between selected loci can also strengthen the effect of hitchhiking on patterns of introgression, with two contrasting outcomes: increasing effective migration rate under strong negative frequency‐dependent selection, and decreasing it with strong selection against hybrids. This implies that the genomic architecture of loci involved in both self‐incompatibility and reproductive isolation will determine patterns of gene exchange in hybrid zones. Self‐incompatibility system may also influence the interaction between hybrid inviability and the rare allele advantage. Sporophytic self‐incompatibility (simulated here) is more mate‐limited than gametophytic self‐compatibility, where the incompatibility reaction is determined by the haploid pollen genotype (Castric & Vekemans, 2004). Consequently, negative frequency‐dependent selection may be stronger in sporophytic systems, so that the patterns illustrated here represent the clearest case for the effect of self‐incompatibility on gene flow in hybrid zones.

#### Unilateral incompatibility and the SI** × **SC rule

While self‐incompatibility can facilitate gene flow between SI populations, it can also limit gene flow between SI–SC species pairs. Specifically, SI styles reject pollen from SC species, while the reciprocal cross often succeeds, in a form of unilateral incompatibility (UI) known as the SI × SC rule (Lewis & Crowe, 1958) (Fig. 1b). In addition to the classic evidence for this rule (e.g. Lewis & Crowe, 1958), recent studies have shown that this pattern of cross‐incompatibility holds in the wild tomato clade (Solanaceae, Baek *et al*., 2015), and in the mustards (Brassicaceae, Li *et al*., 2018). However, studies in additional taxa are needed for a more thorough characterization of the occurrence of this ‘rule’.

We suggest that rejection of interspecific pollen is a nearly unavoidable pleiotropic consequence of most SI mechanisms. The mechanistic basis of the SI × SC rule is best understood in wild tomatoes, which reject self‐pollen via the collaborative nonself‐recognition system (Kubo *et al*., 2010). Here, SC pollen in SI × SC crosses is rejected because a loss‐of‐function mutation in the gene *Cullin* prevents pollen from fully detoxifying stylar SRNases (Li & Chetelat, 2010). Pollen from SC populations with loss of function *Cullin* variants are rejected by SI plants, but SC pollen with a functional *Cullin* are not (Markova *et al*., 2016). As such, the SI × SC rule does not appear immediately, but rather requires sufficient divergence such that the *Cullin* function breaks down, an observation consistent with the fact that some SC populations/accessions can fertilize SI plants (Hogenboom, 1975). The mechanism of UI is less well understood in mustards (Brassicaceae), but is likely caused by the same pollen rejection pathway responsible for rejecting self‐pollen (Li *et al*., 2018). Finally, in the Polemoniaceae, the strong correlation between self‐specific and interspecific incompatibility is likely due to the pistil‐controlled strength of pollen adherence to the stigma (Roda & Hopkins, 2019). Despite differences in the underlying mechanism of UI, this combination of divergent mating systems (SI × SC) should have similar consequences for hybridization.

We predict that the SI × SC rule will decrease the extent of gene flow and will prevent the introgression of SC cytotypes into SI populations. The seven SI × SC species pairs in our dataset are consistent with this prediction. Only one such species pair was classified as having a high level of gene flow (Fig. 3c), three had low gene flow, one had very low gene flow, and the remaining two had a low level of gene flow that varied among populations (Fig. 3c). While asymmetries in gene flow between SI × SC species pairs requires more study – all four of the species‐pairs for which asymmetry was characterized demonstrated clear asymmetries in gene flow (Fig. 3d) and the direction of this asymmetry was variable. This variability in the direction of gene flow highlights a key difference between asymmetry in cross direction (i.e. which species is the pollen donor during initial hybridization) and asymmetry of introgression (i.e. which species introgresses more foreign ancestry).

## Self‐compatibility

While self‐incompatible plants are incapable of self‐fertilization, the extent of cross‐fertilization (quantified as outcrossing rate – *t*_m_) can vary dramatically in plants capable of self‐fertilization. Although *t*_m_ is continuous, we follow the tradition in the field (e.g. Goodwillie *et al*., 2005) of using *t*_m_ to categorize species as selfing (*t*_m_ < 0.2), outcrossing (*t*_m_ > 0.8) and mixed‐mating (0.2 < *t*_m_ < 0.8). Assessing this spectrum allows us to investigate the effects of variation in selfing and outcrossing distinctly from highly outcrossing species (*t*_m_ close to 1) with genetically based self‐incompatibility systems.

### The importance of selfing‐outcrossing and genetic load in hybrid zones

In addition to directly impacting the opportunity for gene exchange due to pleiotropic effects of self‐(in)compatibility on interspecific‐(in)compatibility, the altered genetic load that evolves under different systems can shape the extent and direction of introgression and the structure of hybrid zones. Mating system has two distinct effects on the nature of genetic load, both of which can affect introgression. First, we expect a large number of highly deleterious and recessive rare variants in outcrossers, and the purging of such variation in selfers. Second, we expect numerous mildly deleterious mutations to achieve high frequency in selfers whose reduced effective population size cannot efficiently remove such variation. These features generate strong predictions about the direction and extent of introgression, as well as the shape of hybrid zones, and enable tests of the hypothesis that genetic load can shape hybrid zones (Bierne *et al*., 2002; Kim *et al*., 2018).

The presence of the recessive load in outcrossers, and the historical ‘purging’ of such mutations in selfers (Barrett & Charlesworth, 1991; Husband & Schemske, 1996) will shape patterns of introgression. Specifically, the selfed progeny of F_1_s between selfers and outcrossers will suffer low fitness and this will distort transmission in favour of ancestry from the selfer (Fig. 1). By exposing this recessive load from an outcrosser upon selfing, F_1_s could limit the introgression of ancestry from the outcrosser into the selfer. We therefore expect a deficit of unimodal hybrid zones between selfers (*t*_m_ < 0.2) and outcrossers (*t*_m_ > 0.2) – as such unimodal zones will only be maintained if one of the species is ecologically displaced. The absence of unimodal hybrid zones in all SC pairs with different mating systems (SC‐OC × SC‐S) is consistent with this idea (Binomial test – zero successes of seven trials, two‐tailed *P *=* *0.0156). The fact that all seven cases also exhibit asymmetric introgression from selfing to outcrossing species further support the hypothesis that exposure of genetic load in an outcrossing species can prevent its introgression into a selfing species.

Furthermore, because selfers generally have a reduced effective population size and less‐effective purifying selection than outcrossers (e.g. Qiu *et al*., 2011; Arunkumar *et al*., 2015), a larger number of mildly deleterious alleles can drift to higher frequency in selfers than in outcrossers. After introgression into an outcrossing population, these mildly deleterious mutations will be selectively removed, and take with them the linked ancestry derived from the selfer. This argument is not unique to selfer‐outcrossing pairs, but rather relates to any species pairs with different effective population sizes (Kim *et al*., 2018). If this load is severe, it (perhaps in concert with Bateson–Dobzhansky–Muller incompatibilities (BDMIs)) will result in low‐fitness early generation hybrids, which are consistently created by hybridization and removed by selection, resulting in trimodal hybrid zones. When such load (and/or BDMIs) is less severe, we expect selfing‐derived ancestry to introgress into outcrossing populations and be slowly removed by natural selection in these outcrossing populations, resulting in a bimodal hybrid zone. In such cases, we predict genome‐wide selection against the mildly deleterious variants residing on selfing‐derived ancestry, a prediction consistent with the negative correlation between recombination rate and *Mimulus nasutus* (selfing) ancestry in the outcrossing SC species, *M. guttatus* (Brandvain *et al*., 2014). Finally, the nature of the load in selfing populations – numerous mildly deleterious mutations at high frequency – can shape the composition of hybrid zones between selfing populations. Secondary contact between selfing populations allows the removal of their unique mildly deleterious mutations (Harkness *et al*., 2019), which could result in unimodal hybrid zones, as observed in three of the four SC‐S × SC‐S species pairs (Fig. 3b). However, both ecological differences between populations and epistatic combinations that readily arise in selfing populations may act as an additional barrier to gene flow, resulting in bimodal or trimodal hybrid zones.

### Alternative and/or complementary explanations for asymmetries in introgression

While differences in genetic load may result in an absence of unimodal hybrid zones and asymmetric introgression from selfers to outcrossers, connecting this pattern to process remains challenging. Initially, alternative mechanisms may generate similar patterns in modality and asymmetry in hybrid zones. For example, the higher effective recombination rates in outcrossers could allow neutral alleles from selfers to escape their maladaptive background, thereby avoiding removal by selection. Similarly, the lower effective recombination rate in selfing lineages may prevent neutral or adaptive outcrossing‐derived ancestry from escaping its maladaptive background in selfers. Additionally, the type of selfing and pollen dispersal capacity will influence the direction of introgression. Unidirectional introgression from outcrossers to selfers is consistent with ‘prior‐selfing’ given that outcrossers have no opportunity to fertilize selfers. Yet, greater pollen export in outcrossers could result in the opposite prediction. Similarly, the dominance of mating system traits could also modify the extent and direction of introgression. For example, if F_1_s do not self, then the predictions concerning the recessive load are irrelevant for the direction of introgression. In summary, the observed asymmetries in gene flow are consistent with simple predictions from the nature of the genetic load, however, whether this explanation, or other alternative mechanisms drive these patterns requires additional empirical and theoretical investigations (see Hu (2015) for one such theoretical treatment).

### Mixed mating and variation in selfing: the costs vs benefits of outcrossing in hybrid zones

Mixed mating describes hermaphroditic plants that reproduce through both self‐ and cross‐fertilization. This strategy is widespread, and, while obtaining unbiased estimates of the frequency of mixed‐mating is difficult, one previous study estimated that approximately one quarter of flowering plant taxa had intermediate outcrossing rates (i.e. selfing rates between 0.2 and 0.8; Igic & Kohn, 2006). In contrast to predominantly selfing or obligate outcrossing taxa, mixed‐maters often display substantial variation in outcrossing rates among populations and species (Whitehead *et al*., 2018). In the context of hybrid zones, this variation provides considerable scope for differences in the relative production of selfed, outcrossed and hybrid offspring.

Knowing mating patterns in hybrid zones of mixed‐maters (0.2 < *t*_m_ < 0.8) is crucial for understanding the importance of pre‐zygotic factors in regulating variation among populations and species. This is often quantified using outcrossing rates inferred through indirect genetic estimates (Ritland, 2002) or directly through pedigrees or paternity analysis (e.g. Field *et al*., 2011). Despite the widespread measurement of outcrossing rates (*t*_m_) in the plant mating system literature, surprisingly this has rarely been quantified in plant hybrid zones (Rieseberg *et al*., 1998). The frequency of self‐pollination is often implicated as an important mediator of gene flow between populations. This is because higher selfing limits the availability of ovules for outcross pollination (Lloyd, 1979) from both conspecific and interspecific sources (Fishman & Wyatt, 1999). As such, the type of selfing (prior, competing and delayed; Lloyd, 1979) in mixed mating systems is an important feature that likely mediates opportunities for hybridization (Goodwillie & Weber, 2018). In a study of several *Centaurium* species, Brys *et al*. (2016) reported that prior selfing generates a stronger reproductive barrier to hybridization compared with other forms of selfing. This study suggests that delayed selfing generates a weaker barrier, and may provide the greatest opportunities for hybridization when interspecific pollen is readily available. In addition, strongly divergent outcrossing rates may generate the conditions for faster pollen tube growth rates in the predominantly outcrossing species (Mazer *et al*., 2018) (Fig. 1). Whether SC–SC systems are more sensitive to ecological and demographic factors that influence the availability of self, outcross (intraspecific) and interspecific pollen requires more detailed studies of mating patterns in replicate hybrid zones.

Hybrid frequency is often quantified in hybrid zones (*n* = 59 of 133 studies), yet only a few studies have quantified the local factors that may drive mating patterns. For example, across 18 hybrid zones between *Eucalyptus aggregata* and both *E. viminalis* and *E. rubida* (insect pollinated trees), the relative abundance of parental species was a significant predictor of the frequency of hybrid seed (Field *et al*., 2008). Using paternity analysis for detailed characterization of local mating patterns indicated that, in addition to individual flowering synchrony, the same relations exist at a local scale within populations (Field *et al*., 2011). The relative abundance of parental species has also been shown to be important for hybridization in wind‐pollinated trees (e.g. Lepais *et al*., 2009; Lagache *et al*., 2013). This demonstrates the local demographic context of individual plants can be important in regulating the frequency of hybridization in mixed‐maters across species with very different pollen vectors.

The formation of hybrid zones between mixed maters is likely to result in a diverse set of outcomes depending on the specific set of barriers, fitness of inbred vs hybrid offspring and the ecological and demographic context in which the population resides. This is reflected in the observation that hybrid zones between mixed maters exhibited similar frequencies of mode (bimodal, trimodal, unimodal), rates of gene flow and the presence of bilateral and asymmetrical gene flow (Fig. 3). Consequently, the genomic consequences of hybridization between mixed maters are also likely to be highly idiosyncratic. Unlike SI × SC systems, where there are drastic differences in local recombination rates or effective population size, for mixed maters we may expect similar dynamics of admixture and introgression blocks along genomes. Due to variation in the type of selfing, we may ask if mixed maters are more sensitive to local demography and phenology, showing stronger relations between these factors and rates of gene flow? Are mixed‐ mating systems more prone to genetic or demographic swamping, or conversely, adaptive introgression? Answering these questions would require more detailed comparisons of genomic outcomes in relation to demography across replicate hybrid zones with variation in mixed mating.

## Dioecy and sexual system variation

### Can sexual system variation promote asymmetric gene flow?

Sexual system variation may influence hybridization through differences in sex allocation, colonization ability or sex ratios in species pairs with different sexual systems. Differences in sex allocation dynamics between plant species with separate sex (dioecious) vs co‐sexual populations (Charnov, 1982) may contribute to asymmetric gene flow following hybridization. The trade‐off in resource allocation between male and female reproductive functions in hermaphrodites (Goldman & Willson, 1986) may result in lower pollen or seed production relative to males and females. These differences in reproductive traits would be even more pronounced in predominant selfers compared to outcrossers (Lloyd, 1987), and may cause asymmetric introgression in hybrid zones from dioecious or gynodioecious populations into co‐sexual ones (Fig. 1, section 3a). Consistent with this prediction, Buggs & Pannell (2006) found highly asymmetric hybridization by pollen swamping in wind‐pollinated *Mercurialis annua* from a dioecious diploid lineage into a monoecious hexaploid lineage. The dioecious *M. annua* plants produce more pollen than the monoecious facultative selfers leading to a rapid displacement of the hybrid zone. Similar results were found by Wallace *et al*. (2011) in a hybrid zone between *Schiedea menziessi* (hermaphrodite SC‐OC) and *S. salicaria* (gynodioecious SC‐G), where the numerous wind‐dispersed pollen grains of *S. salicaria* increased pollen transfer efficiency into its more selfing, and not wind‐dispersed, sister species.

### Dioecy and the evolution of reproductive isolation

Although dioecy has evolved in few plant species (*c.* 6% of flowering plants), it is phylogenetically widespread (Renner & Ricklefs, 1995) and hybridization has been observed between D taxa (*n* = 9 species pairs in our analysis). Species with separate sexes may experience intra‐genomic conflict for traits that have different optima in females vs males, possibly leading to arms‐race evolution (Bonduriansky & Chenoweth, 2009). This evolutionary change may promote the fixation of BDMIs (Johnson, 2010) between D species, increasing reproductive barriers. Accordingly, D species may consistently evolve stronger post‐zygotic barriers compared to other sexual systems (Fig. 1, section 3b). In our comparative analysis, the presence of intrinsic incompatibilities was reported in 78% of the D × D species pairs (seven of nine) compared to between 22 and 35% for the other systems (SC × SC: 23 of 67; SI × SI: 10 of 42; SI × SC: two of seven), representing a significant excess of incompatibilities in D × D pairs compared to the rest of the data set (*χ* ^2^ = 7.02, df = 1, *P *=* *0.008). This preliminary data is consistent with the hypothesis that D species are more likely to exhibit intrinsic incompatibility. However, it is also difficult to exclude ascertainment bias with only a small sample size available. Therefore, more work is required to test the generality of this trend and to understand the mechanisms and drivers of greater intrinsic genetic incompatibility between species with separate sexes.

The presence of sex chromosomes may further reinforce genetic conflicts, as they are particularly susceptible to segregation distortion (Frank, 1991). This is because recombination between the sex chromosomes is limited, and therefore these two chromosomes are constantly involved in a conflict for segregation. Since segregation distorters and their suppressors co‐evolve independently within separate lineages, their interaction in hybrids may be a source of BDMIs. Accordingly, sex chromosomes are involved in one of the strongest pattern characterizing speciation in animals (Coyne & Orr, 2004): ‘Haldane's rule’, whereby hybrids of the heterogametic sex express more BDMIs than hybrids of the other sex. Moreover, an excess density of genes causing hybrid sterility or inviability on the sex chromosomes of animals (the ‘large X effect’, Coyne & Orr, 2004) also highlights their importance for reproductive isolation. However, little work has been done on the role of sex chromosomes in plant speciation. This is mainly due to their rarity and that plant sex chromosomes are commonly nondegenerated (Ming *et al*., 2011), making them harder to detect. Nevertheless, variation in plant sex chromosomes – from homomorphic sex chromosomes (e.g. in *Populus* and *Salix*) to heteromorphic sex chromosomes with Y (or W) degeneration (e.g. in *Silene*) (Ming *et al*., 2011) – provides the unique opportunity to examine how different types of sex chromosomes may act as a barrier to interspecific gene flow. Overall we predict genetic incompatibility between D species and their relatives to be highest for heteromorphic, degenerated sex chromosomes, lower for homomorphic sex chromosomes, and lowest for autosomal sex determination (Fig. 1).

Although plants have the potential to shed light on these poorly understood topics, *Silene* and *Rumex* are currently the only two genera where these questions have been investigated. Brothers & Delph (2010) found evidence of Haldane's rule in crosses between three closely‐related *Silene* species with young sex chromosomes (*S. latifolia*,* S. dioica* and *S. diclinis*). Contrary to its close relatives, the Y chromosome of *S. latifolia* has undergone degeneracy leading to the upregulation of X‐linked gene expression in males. This species‐specific dosage compensation mechanism has been proposed to promote the evolution of sex‐linked BDMIs between *S. latifolia* and *S. dioica* (Filatov, 2018). For *R. hastatulus*, hybridization between two chromosome races provided evidence of Haldane's rule for both male fertility and rarity (Kasjaniuk *et al*. 2019). Taken together, these results demonstrate that the sex chromosomes in *Silene* and *Rumex* likely act as a major barrier to gene flow between hybridizing species, and emphasizes the need for similar investigations in other dioecious plants.

## Conclusions and future directions

Hybrid zones have long fascinated evolutionary biologists by providing a window into the speciation process (Harrison, 1990). Although much of this work has progressed in isolation from the study of plant mating systems, we demonstrate how bridging these fields can reveal new insights into both barriers to gene flow and the evolution of mating systems. The influence of mating system on hybridization can also be viewed as a ‘two‐way street’ where hybridization may influence the evolution of mating and sexual systems themselves, with unique outcomes for each mating system type. For example, for self‐incompatibility, theory provides insight into the conditions associated with evolution of novel SI types (e.g. Gervais *et al*., 2011; Bod'ová *et al*., 2018) yet observed S allele diversity (Lawrence, 2000) often exceeds theoretical estimates. Hybridization among species may enhance diversification, with novel S haplotypes evolving in separate species and subsequently being exchanged among species via introgression (Castric *et al*., 2008). For mixed maters, hybridization may affect mating system evolution by reinforcing selfing to avoid wasting gametes and resources on hybrid progeny (Fishman & Wyatt, 1999; Goodwillie & Ness, 2013). This may involve increased selfing (e.g. Briscoe Runquist & Moeller, 2014) and/or the evolution of floral traits associated with increased selfing including lower pollen production, herkogamy and smaller floral displays (e.g. Smith & Rausher, 2007; Wright *et al*., 2013; Brys *et al*., 2016). Finally, hybridization of divergent SC and D populations can promote the evolution of rare ‘mixed’ sexual systems such as androdioecy (males and hermaphrodites; *Mercurialis annua*, Obbard *et al*., 2006) and trioecy (males, females and hermaphrodites; *Sagittaria latifolia*; Yakimowski & Barrett, 2016). Going forward, more estimates of hybridization for mixed reproductive systems are required to determine how frequently they represent a phenotypic signature of hybridization.

Our comparative study suggests that the type of mating systems that interact can alter the strength of barriers and asymmetries in gene flow. At one end, in SI × SI systems, rare allele advantage may facilitate introgression and hinder population divergence. By contrast, transitions to selfing and secondary contact between SI × SC systems can generate strong and asymmetrical barriers. Consequently, mating system diversity has the potential to directly influence the rate of gene flow between evolutionary lineages and regulate the tempo of speciation. Yet, this study also highlights that currently available data is insufficient to specifically test these hypotheses. We therefore conclude by identifying several open questions (and the data required to answer these) (Box 1) that could strengthen our understanding of the interaction between hybridization and mating systems. In addressing these questions, novel insights will likely come from integrating theory with in‐depth genetic analyses of hybrid zones. By using this integrated approach we can compare how mating system contributes to patterns of gene flow and the interaction of divergent genomes over short and long evolutionary timescales.

## Author contributions

MP and DLF developed the research. MP, DLF and EC collected the comparative data. DLF, MP, YB and CF analysed the comparative data. MP, NHB, TD and DLF developed and ran the simulation model. DLF, MP, CF, YB and SY prepared the figures. MP, DLF, YB, CF and SY wrote the first draft of the manuscript. All authors reviewed and edited the final version of the manuscript.

## Supporting information

Please note: Wiley Blackwell are not responsible for the content or functionality of any Supporting Information supplied by the authors. Any queries (other than missing material) should be directed to the *New Phytologist* Central Office.

**Methods S1** Comparative analysis methods.
**Methods S2** Self‐incompatibility model.
**Table S1** The potential influence of different mating/sexual systems on patterns of gene flow and pre‐mating and post‐mating pre‐zygotic and post‐zygotic reproductive isolating barriers.

## Data Availability

The comparative data set is available from Dryad at doi: https://doi.org/10.5061/dryad.dm7cs86.
