# Supplementary material for: Mating system variation in hybrid zones: facilitation, barriers and asymmetries to gene flow
Source: New Phytol. 2019 Oct 9;224(3):1035–47. doi: 10.1111/nph.16180 (PMC6856794; doi:10.1111/nph.16180)
Supplement: Supplementary file 1 — Methods S1 Comparative analysis methods. Methods S2 Self‐incompatibility model. Table S1 The potential influence of different mating/sexual systems on patterns of gene flow and pre‐mating and post‐mating pre‐zygotic and post‐zygotic reproductive isolating barriers. [file NPH-224-1035-s001.pdf]

New Phytologist Supporting Information

Article title: Mating system variation in hybrid zones: Facilitation, barriers and asymmetries to gene flow

Authors: Melinda Pickup, Yaniv Brandvain, Christelle Fraisse, Sarah Yakimowski, Nicholas H. Barton, Tanmay Dixit, Christian Lexer, Eva Cereghetti and David L. Field

Article acceptance date: 19 August 2019

The following Supporting Information is available for this article:

Table S1. The potential influence of different mating/sexual systems on patterns of gene flow and pre- and post-mating pre-zygotic and post-zygotic reproductive isolating barriers

Methods S1. Comparative analysis methods

Methods S2. Self-incompatibility model

Table S1. The potential influence of different mating/sexual systems on patterns of gene flow and pre- and post-mating pre-zygotic and post-zygotic reproductive isolating barriers. SI – genetically based self incompatibility, SC – self compatible, SC-S – predominant selfer (t_m < 0.2, selfing syndrome), SC-OC – can self, but outcrossing rates vary from mixed maters (t_m = 0.2 – 0.7) to predominant outcrossers (t_m > 0.8), D – Dioecious, D-CR – Dioecious with sex chromosomes, G – Gynodioecious, BDMIs – Bateson-Dobzhansky-Muller incompatibilities. SRNase – S-locus (S) RNase-mediated self-incompatibility mechanism found in Solanaceae, Rosaceae and Plantaginaceae (see Fujii *et al.*, 2016). t_m = outcrossing rate.

Mating system	Gene flow	Pre-mating prezygotic	Post-mating prezygotic	Post-zygotic
**Self-incompatible (SI)**<br>Species with a genetically based system that prevents selfing and mating among relatives	**SI x SI**: Facilitate gene flow due to S alleles<br><br>**SI x SC**: Asymmetrical gene flow between SI and SC species		SI x SC: Relic SRNase genes (non-self recognition SI) may be involved in isolating barriers	
**Self-compatible (SC)**<br>Species capable of self-fertilization: can range from predominate selfing (**SC-S**; t_m <0.2) to mixed mating (**SC-OC**; t_m 0.2 - 0.7) to predominate outcrossing (**SC-OC**; t_m >0.8).<br><br>Consequences for barriers and gene flow will depend on the amount of sefling and differences in selfing and outcrossing in the species pair:<br>**SC-S x SC-S** (both highly selfing)<br>**SC-OC x SC-OC** (both with some degree of outcrossing)<br>**SC-S x SC-OC** (one highly selfing, the other outcrossing)	**SC-OC x SC-S**: Outcrossing taxa more successful at pollen transfer: asymmetrical gene flow Outcrosser→ Selfer	**SC-S**: Lower pollinator visitation for highly selfing taxa due to floral changes associated with selfing syndrome<br><br>**SC-OC x SC-OC**: for mixed maters demographic context can influence outcrossing rates and thus rates of interspecific gene flow	**SC-S**: Conspecific pollen precedence greater in highly selfing species<br><br>**SC-OC x SC-S**: Differential pollen tube growth rates. Outcrosser pollen more competitive. Pollen more competitive in species with lower correlated paternity<br><br>**SC-OC x SC-OC**: for mixed maters a higher proportion of selfed pollen reduces overall competitiveness of conspecific pollen, can result in higher success of interspecific pollen	Higher selfing generates stronger reproductive isolation and reinforcement<br><br>Highly selfing species may have greater BDMIs resulting in hybrid breakdown in F_2 and later generations<br><br>Asymmetric incompatibilities due to imprinted loci.<br><br>Mildly deleterious mutations that accumulate in small N_e selfers prevent introgression of outcrossing ancestry<br><br>Higher inbreeding reduces conspecific offspring fitness compared to hybrids. Balance depends on the costs of inbreeding vs. hybrid breakdown
**Dioecious (D)**<br>Species with separate sexes (male and female reproductive organs in separate individuals). Species may have **sex chromosomes (D-CR)**	**D x SC-S or D x SC-OC:** Asymmetric pollen/ovule production, with males and females >hermaphrodites. This difference is			Sex chromosomes lead to stronger interspecific barriers. Likely to be stronger in species with heteromorphic compared to

	likely greatest for D x SC-S species pairs<br><br>**SC-S/SC-OC x D:** Asymmetries in gene flow from SC to D species due to the ability of a single SC individual to sexually reproduce upon colonization			homomorphic sex chromosomes<br><br>Sex chromosomes may lead to greater BDMIs<br><br>**D x SC-S or D x SC-OC:** Differences in cytoplasmic-nuclear interactions may cause asymmetries in hybridization
**Gynodioecious (G)**<br>Individuals are either female or hermaphrodite (male and female reproductive organs)		**G x SC-S or G x SC-OC:** Greater pollen production in G may result in asymmetries in hybridization		**G x SC-S or G x SC-OC:** Differences in cytoplasmic-nuclear interactions may cause barriers and asymmetries

Methods S1. Comparative analysis methods

Species pairs included in our analysis were identified using Abbott (2017), Lowry et al. (2008) and from literature searches using the key words ‘hybridization’ and ‘plant’. For each species pair, an identified (active) hybrid zone was required to be included in the study. For all taxa we classified mating system and collected life history traits including pollen vector (biotic, abiotic and pollinator type) and growth form (tree, shrub, herbaceous). Traits and mating system type were identified using published literature sources and, for some cases, online species descriptions. Mating system classifications were: self-incompatible (SI), self-compatible (SC), dioecious (D), Gynodioecious (G), Androdioecious (A) and Trioecious (T) (for a description of each mating system type see Table S1). In our paper we refer to populations containing all three sex phenotypes (females, hermaphrodites, males) as trioecious; trioecy and subdioecy are used interchangeably to refer to this sexual system, and for consistency we use trioecy throughout. Androdioecy is a relatively rare sexual system containing hermaphrodites and males. Gynodioecy refers to populations containing hermaphrodites and females.

We further classified species capable of self-fertilization (self-compatible, SC) into predominantly selfing (SC-S) and predominantly outcrossing (including mixed maters, SC-OC) based on outcrossing rates (t_m) (where available, using also Goodwillie *et al.*, 2005; Moeller *et al.*, 2017) and descriptions from the literature (SC-S: t_m <=0.2, SC-OC: t_m >0.2). For dioecious species, information on sex chromosomes was obtained from Ming et al. (2011).

We used hybrid zone mode (unimodal, bimodal and trimodal) to describe the general genotypic composition of each hybrid zones and provide broad information on the strength of reproductive barriers (see also Fig. 2). The type of genetic marker used (Allozymes, RFLPs, AFLPs, SSRs, SNPs) and number of loci involved (4-1000's) varied considerably between studies. Therefore, information on the mode for each species pairs was based on the type of hybrids identified, admixture categorization and descriptions of hybrid frequency and distribution. A hybrid zone was classified as unimodal if a range of hybrid admixture types was present (parents, F1s, F2s, backcrosses and/or later generation hybrids). A hybrid zone was bimodal if there were predominantly parental genotypes and a low frequency of hybrids. While trimodal hybrid zones consisted predominantly of parents and F1 hybrids.

Following Abbott (2017) we categorized gene flow into four categories: very low, low, high and variable. There are, of course, caveats to any single approach/measure for classifying gene flow. Moreover, the diversity of marker and analysis types across the 127 studies precluded the use of a single quantitative number to classify gene flow (see main text). To categorize each species pair, we used information on the frequency of hybrids and backcrosses (numbers of each hybrid class (F1s, F2s and backcrosses to each parental type) based on STRUCTURE, NewHybrids, Hybrid index) and models of gene flow (IM models such as Migrate, IMa2, Lamarc). Where available F_{ST} (between populations adjacent to the hybrid zone) was also used as an indicator of gene flow between taxa (and $N_em = 1 - F_{ST}/4 * F_{ST}$ (Wright, 1931; used in Rieseberg *et al.*, 2004)), although F_{ST} was interpreted with caution and does not equal gene flow. Allocation to gene flow categories was first based on hybrid frequency and the presence of backcrosses. Then, interpretations/conclusions of gene flow from individual studies, Abbott (2017) (supporting information Table S7) and F_{ST} values (where available) were considered in allocating species pairs to one of four categories. If there was insufficient information, a gene flow category was not assigned:

Very low = very few hybrids observed and backcrosses and advanced generation hybrids absent (or very low frequency). Generally high F_{ST} ($F_{ST} > 0.3$)

Low = low frequency of hybrids, backcrosses and advanced generation hybrids. Generally high F_{ST} ($F_{ST} > 0.2$)

High = high frequency of hybrids and the presence of backcrosses and advanced generation hybrids. Generally low F_{ST} ($F_{ST} < 0.2$)

Variable = patterns of gene flow varied among hybrid zones (applicable when multiple hybrid zones were studied, often in relation to ecological gradients).

This information (either one or multiple quantitative measures) was available for 74 of the 127 studies (n = 127 with information on mating system for both taxa). As stated above, for the 53 studies without this quantitative information, we used information from Abbott (2017) (supporting information Table S7) and conclusions from the original study to make the classification. We then examined gene flow category in relation to mating system with, and without, these studies to examine their effect on the overall distribution of gene flow categories across the mating system types. We found a very similar distribution of gene flow categories for each mating system type for our conservative approach that included only studies with quantitative estimates (n = 74, Figure A) compared to including studies without quantitative estimates (n = 127, Figure 2c). Moreover, we found our classifications were associated with hybrid frequency and F_{ST} (see Figure B(a) and B(b)) and studies with higher F_{ST} generally had lower hybrid frequency (see Figure C).

For each of these gene flow categories (very low, low, high and variable), we classified if the gene flow was asymmetric (asymmetries = yes), bilateral (asymmetries = no) or no information (not stated). For asymmetric gene flow we recorded the direction of gene flow between parental taxa. Asymmetries in gene flow were identified in each study using the proportion of each backcross type.

Information on the presence/absence of post-zygotic intrinsic incompatibilities was collected from each study using Abbott (2017) Supporting Information Table S6 and by cross-checking for evidence of intrinsic incompatibilities in each individual study. Studies with the presence of post-zygotic incompatibilities was allocated (1), absence/no evidence (0) and not sufficient information/not stated (not stated).

Statistics: All statistical analyses were conducted in R. All analyses called for $\chi 2$ contingency or goodness of fit tests. However, in some cases, small numbers for expectations violated assumptions of tests, and we therefore generated simulation or permutation-based p-values. We present our R code below.

```
# Load packages
library(tidyverse)
library(infer)
# Load data
HZ_database <-read.csv(file = "HZmatingSystem_Rimport.csv")

#Summarize  mating system counts
```

```
HZ_database %>% filter(Mating_system_BOTH_TAXA != "No info found") %>%
  group_by(Mating_system_BOTH_TAXA) %>%
  summarise(prop = n()) %>%
  mutate(prop = prop / sum(prop))
```

	Mating system BOTH TAXA	prop
## 1	And-Tri	0.00787
## 2	D-D	0.0709
## 3	SC-Gyn	0.00787
## 4	SC-SC	0.528
## 5	SI-SC	0.0551
## 6	SI-SI	0.331

## Analysis for mating system combinations in hybrid zones

```
HZ_database %>% filter(Mating_system_BOTH_TAXA == "SC-SC") %>%
  group_by(Mating_system_BOTH_TAXA_OUTCROSSING) %>%
  summarise(count = n())  %>%ungroup() %>%
  mutate(tot = sum(count))
```

	Mating_system_BOTH_TAXA_OUTCROSSING	n (of 67 total)
## 1	SC-OC_SC-OC	55
## 2	SC-OC_SC-OC	1
## 3	SC-OC_SC-S	4
## 4	SC-S_SC-OC	2
## 5	SC-S_SC-OC	1
## 6	SC-S_SC-S	4

```
expect1 <- tibble(count = c(4, 7, 56), type = c("sxs","outXs","outXout")) %>%
  mutate(expect.prop = dbinom(x = 0:2,size = 2,prob = (56 + 7/2)/sum(count) ),
         expect.n    = expect.prop * sum(count))
obs.chi2 <- expect1 %>%
  mutate(chi2 = (expect.n - count)^2 / expect.n ) %>%
  summarise(chi2 =sum(chi2)) %>% pull()
expect <- expect1 %>%
  select(expect.n) %>%
  pull()
### p-value
as_tibble(data.frame(t(rmultinom(n = 100000000, size = 67, prob =
                        dbinom(x = 0:2, size = 2, prob = (56 + 7/2)/67 ))))) %>%
  rename(SxS = X1, OxS  = X2, OxO = X3) %>%
  mutate(chi2=(SxS-expect[1])^2/expect[1]+(OxS-expect[2])^2/expect[2]+(OxO-expect[3])^2/expect[3])
%>%
```

```
  summarise( p.val = mean(chi2 >= obs.chi2)) %>%
  pull()
## [1] 0.00265572 # p-value
```

The frequency of hybrid zone mode

```
findChi2 <- function(this.tibble){
  this.tibble%>%
    group_by(Mating_system_BOTH_TAXA,Y) %>%
    summarise(n = n()) %>% ungroup() %>%
    spread(key = Y, value = n, fill = 0) %>%
    gather(key = Y, value = n, - Mating_system_BOTH_TAXA)%>%
    group_by(Mating_system_BOTH_TAXA) %>%
    mutate(nms = sum(n)) %>% ungroup() %>%
    group_by(Y) %>%
    mutate(nhz = sum(n)) %>% ungroup() %>%
    mutate(expect = nms * nhz / sum(n)) %>%
    summarise( sum((n - expect)^2 / expect)  )%>%
    pull()
}

matingsysXhzmode <- HZ_database %>%
  filter(Mating_system_BOTH_TAXA != "No info found" & Hybrid_zone_mode_classification != "") %>%
  select(Mating_system_BOTH_TAXA,Hybrid_zone_mode_classification)
table(matingsysXhzmode)
```

	Hybrid_zone_mode_classification				
	Mating_system_BOTH_TAXA	Bimodal	Trimodal	Unimodal	Unimodal_Biomodal_variable
## 1	And-Tri	0	0	1	0
## 2	D-D	2	3	2	0
## 3	SC-Gyn	1	0	0	
## 4	SC-SC	20	12	26	2
## 5	SC-SO	2	2	2	0
## 6	SI-SI	11	3	20	1

```
my.chis <- replicate(10000, findChi2(
  matingsysXhzmode %>% mutate(Y = sample(Hybrid_zone_mode_classification))))
  mean(my.chis >= findChi2(matingsysXhzmode %>% mutate(Y = Hybrid_zone_mode_classification)) )
  ## [1] 0.7986  ### chi2

matingsysXhzmodeReduced <- HZ_database %>%
  filter(Mating_system_BOTH_TAXA != "No info found" & Hybrid_zone_mode_classification != "") %>%
       select(Mating_system_BOTH_TAXA,Hybrid_zone_mode_classification) %>%
       filter(Mating_system_BOTH_TAXA %in% c("SC-SC","SI-SI") ) %>%
       filter(Hybrid_zone_mode_classification != "Unimodal_Biomodal_variable") %>%
```

```
  mutate(Mating_system_BOTH_TAXA = droplevels(Mating_system_BOTH_TAXA),
        Hybrid_zone_mode_classification = droplevels(Hybrid_zone_mode_classification))

chisq.test(table(matingsysXhzmodeReduced))
##
##  Pearson's Chi-squared test
##
## data:  table(matingsysXhzmodeReduced)
## X-squared = 2.7197, df = 2, p-value = 0.2567
```

# Levels of Gene Flow

```
gene.flow.level <- HZ_database %>%
  filter(!Mating_system_BOTH_TAXA %in% c("No info found","And-Tri","SC-Gyn") &
         gene_flow_level != "" & !is.na(gene_flow_level)) %>%
  mutate(gene_flow_high_low = case_when(gene_flow_level == "high" ~ "high",
                                        gene_flow_level %in% c("low", "low_variable", "verylow") ~"low"),
        Mating_system_BOTH_TAXA = droplevels(Mating_system_BOTH_TAXA ))%>%
  select(Mating_system_BOTH_TAXA, gene_flow_high_low )
table(gene.flow.level)
```

		gene_flow_high_low	
	Mating_system	high	low
## 1	D-D	1	8
## 2	SC-SC	21	40
## 3	SI-SC	1	6
## 4	SI-SI	23	18

```
chisq.test(table(gene.flow.level) )
##  Pearson's Chi-squared test
## data:  table(gene.flow.level)
## X-squared = 10.316, df = 3, p-value = 0.01606
```

## Gene flow asymmetry

```
asymA <- HZ_database %>%
  filter(gene_flow_asymm != "" & !Mating_system_BOTH_TAXA %in% c("No info found"))%>%
  mutate(asymm = case_when(gene_flow_asymm %in% c("no","No") ~ "no",
                           gene_flow_asymm %in% c("yes","Yes")~ "yes"),
        Mating_system_BOTH_TAXA = droplevels(Mating_system_BOTH_TAXA)) %>%
  select(asymm, Mating_system_BOTH_TAXA)

asymA %>%   table() %>%  rowSums()
```

	no	yes
## 1	24	49

```
asymB <- asymA %>%
  filter(!Mating_system_BOTH_TAXA %in% c("No info found","And-Tri", "D-D", "SC-Gyn"))%>%
  mutate(Mating_system_BOTH_TAXA = droplevels(Mating_system_BOTH_TAXA))

table(asymB)
##      Mating_system_BOTH_TAXA
## asymm SC-SC SI-SC SI-SI
##   no      9     0    14
##   yes    28     4    12
```

		gene_flow_high_low		
	asymm	SC-SC	SI-SC	SI-SI
## 1	no	9	0	14
## 2	yes	28	4	12

```
chisq.test(table(asymB) )
##  Pearson's Chi-squared test
## data:  table(asymB)
## X-squared = 8.1269, df = 2, p-value = 0.01719
```

## Incompatibilities

```
bdmi <- HZ_database %>%
  mutate(PostZ_Intrinsic_incompatabilites = case_when(is.na(PostZ_Intrinsic_incompatabilites )~0,
                                                       PostZ_Intrinsic_incompatabilites==1~1)) %>%
  mutate(both_dio = Mating_system_BOTH_TAXA == "D-D") %>%
  select(both_dio, PostZ_Intrinsic_incompatabilites)

table(bdmi)
##         PostZ_Intrinsic_incompatabilites
## both_dio  0  1
##    FALSE 88 36
##    TRUE   2  7
```

		PostZ_Intrinsic_incompatabilites	
	Both Dioecious	0 (no)	1 (yes)
## 1	FALSE	88	36
## 2	TRUE	2	7

```
chisq.test(  table(bdmi) )
##  Pearson's Chi-squared test with Yates' continuity correction
## data:  table(bdmi)
## X-squared = 7.0214, df = 1, p-value = 0.008054
```

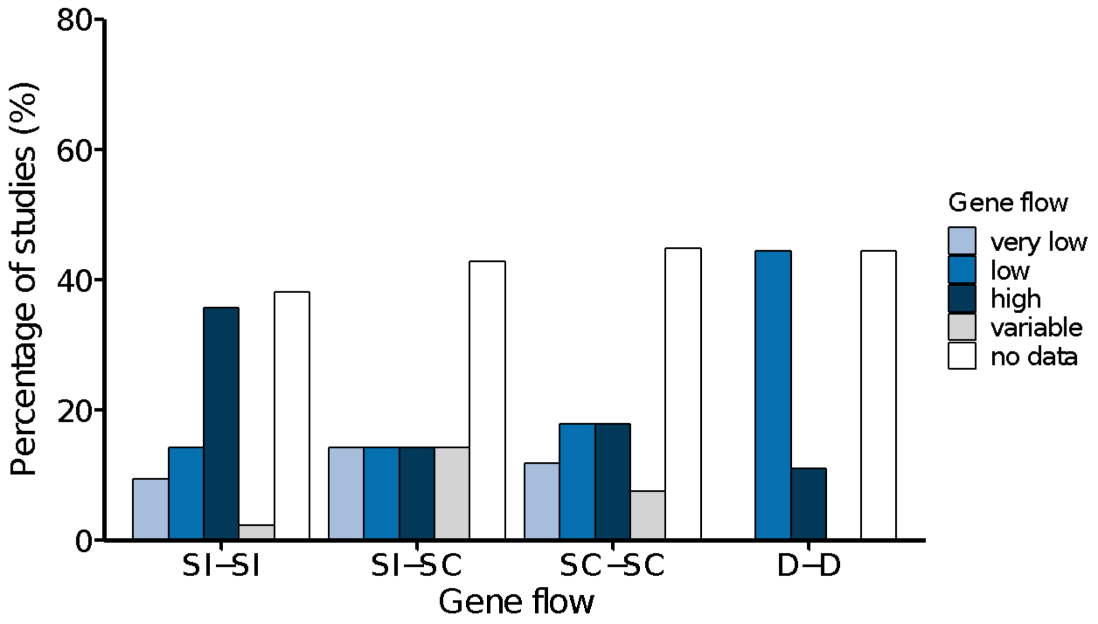

Figure A: The proportion of species pairs categorized as having different levels of gene flow (very low, low, high and variable) for the four main mating system types for the 74 studies with quantitative estimates of frequency of hybrids and backcrosses and/or models of gene flow.

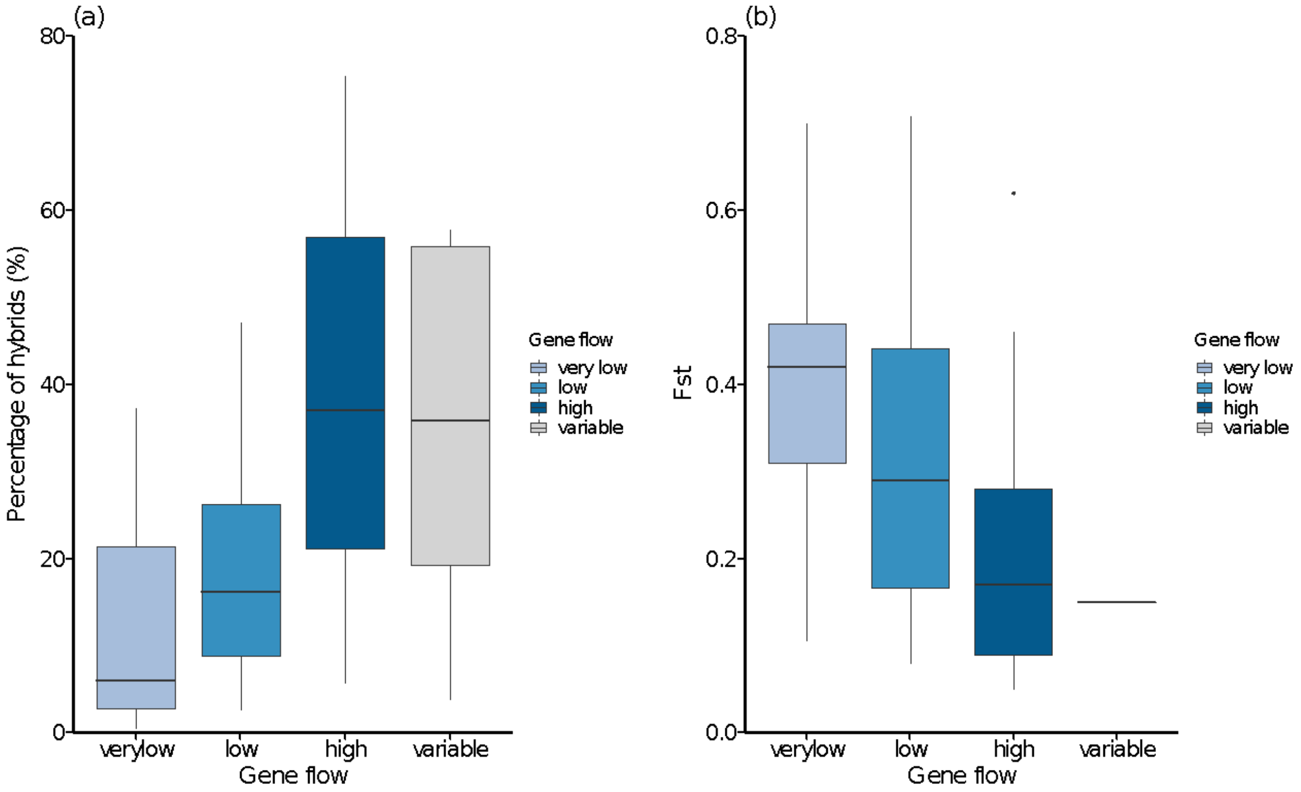

Figure B: The four gene flow categories in relation to the quantitative estimates of (a) percentage of hybrids (*n* = 58 studies) and (b) F_{ST} (*n* = 41 studies). Although there was variation in each category, generally, the very low category had fewer hybrids and higher F_{ST}, the low gene flow category a higher percentage of hybrids and reduced F_{ST}, while the high gene flow category had the highest percentage of hybrids and lowest F_{ST}. For these box plots, upper error bars represents the maximum value, while the lower error bar represents the minimum value in each group.

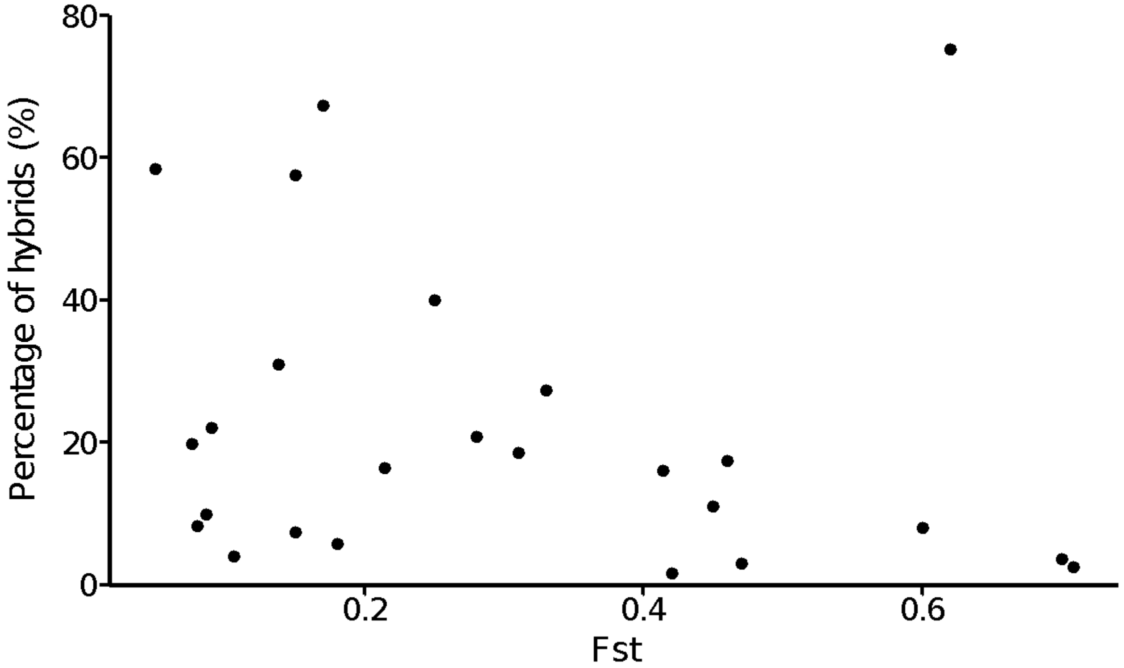

Figure C: The relation between F_{ST} and percentage of hybrids for the 25 studies with both quantitative estimates. This illustrates that, although there was large variation in hybrid percent at low F_{ST}, taxa pairs with higher F_{ST} had a lower percentage of hybrids (with the exception of one study, an orchid species pair: *Orchis milltaris* and *Orchis purpurea*).

References

Abbott RJ. **2017**. Plant speciation across environmental gradients and the occurrence and nature of hybrid zones. *Journal of Systematics and Evolution* **55**: 238–258.

Fujii S, Kubo K, Takayama S. **2016**. Non-self- and self-recognition models in plant self-incompatibility. *Nature Plants* **2**: 16130.

Goodwillie C, Kalisz S, Eckert CG. **2005**. The evolutionary enigma of mixed mating systems in plants: occurrence, theoretical explanations, and empirical evidence. *Annual Review of Ecology, Evolution, and Systematics* **36**: 47–79.

Lowry DB, Modliszewski JL, Wright KM, Wu CA, Willis JH. **2008**. The strength and genetic basis of reproductive isolating barriers in flowering plants. *Philosophical Transactions of the Royal Society of London B: Biological Sciences* **363**: 3009–3021.

Ming R, Bendahmane A, Renner SS. **2011**. Sex chromosomes in land plants. *Annual Review of Plant Biology* **62**: 485–514.

Moeller DA, Runquist RDB, Moe AM, Geber MA, Goodwillie C, Cheptou P-O, Eckert CG, Elle E, Johnston MO, Kalisz S, *et al.* 2017. Global biogeography of mating system variation in seed plants. *Ecology Letters* **20**: 375–384.

Rieseberg LH, Church SA, Morjan CL. 2004. Integration of populations and differentiation of species. *New Phytologist* **161**: 59–69.

Wright S. 1931. Evolution in Mendelian populations. *Genetics* **16**: 0097–0159.

Methods S2. Self-incompatibility model

We simulated two demes, each with 500 individuals for 50 generations using Mathematica. Simulations were based on a sporophytic self-incompatibility system so that the incompatibility reaction is determined by the diploid genotype of each parent. A migration rate (m) of 0.01 per generation was implemented for both seed and pollen, resulting in an actual migration rate of 0.015 per generation (due to its haploid state, the actual migration rate of pollen is 0.005). To assess the influence of S allele diversity and differentiation, we varied the total number of S alleles across both demes (N=8, 16, 24) and the overlap between the demes so that they shared 0%, 50% or 100% of the S alleles. For example, with eight S alleles in total (S_1-S_8), the 0% overlap category would have S_1 to S_4 in deme 1 and S_5 to S_8 in deme 2. In the 50% overlap category, deme 1 would have S_1 to S_6, and deme 2 S_3 to S_8. In the category with 100% overlap, both demes contain all eight S alleles (S_1 to S_8). We predict that the effect of self-incompatibility on effective migration rate would be greatest with fewer S alleles and higher differentiation, because negative frequency dependent selection would be strongest in these situations.

We also varied the strength of selection against hybrids from weak (s = 0.05) to strong (s = 0.2) and very strong (s=0.4) selection. Selection against hybrids was based on heterozygote disadvantage, so that hybrids, which are heterozygous and so contain an allele from each parental type, were selected against. We call this locus the barrier locus. Here we expect stronger selection to reduce effective migration rate. We use effective migration rate as a measure of introgression between the two demes: this was measured at a neutral locus with a recombination rate (r) of 0.1 and 0.5 from the barrier locus. Effective migration rate was calculated using the formula $\Delta P_t = (1 - 2M_e)^t \Delta P_0$ (where ΔP_t is the difference in the frequency of allele P between populations at generation t). This formula is based on the assumption that ΔP_t declines linearly on a logarithmic axis. To minimize errors associated with a non-linear decline in ΔP_t, we calculated the effective migration rate based on the first 25

generations. This also reflects that the effects of the self-incompatibility locus on hybridization dynamics are likely to be greatest in the short-term, after which equilibrium is reached between the two demes. The scripts and Mathematica notebook and code for these simulations can be provided on request.
